# Risk factors for overuse injuries in a cohort of elite Swedish track and field athletes

**DOI:** 10.1186/s13102-021-00297-x

**Published:** 2021-07-08

**Authors:** Andreas Lundberg Zachrisson, Andreas Ivarsson, Pia Desai, Jon Karlsson, Stefan Grau

**Affiliations:** 1 Center for Health and Performance, Department of Food and Nutrition, and Sport Science, University of Gothenburg, Box 300, 405 30 Gothenburg, Sweden; 2 School of Health and Welfare, Halmstad University, Kristian IV:s väg 3, 301 18 Halmstad, Sweden; 3 Department of Orthopaedics at Institute of Clinical Sciences, Sahlgrenska Academy, University of Gothenburg, Göteborgsvägen 31, 431 80 Mölndal, Sweden

**Keywords:** Overuse injuries, Track and field, Risk factors, Biomechanics

## Abstract

**Background:**

Most injuries in track and field are caused by overuse with conflicting reports concerning the underlying mechanisms. The purpose of this study was to evaluate how biomechanical and clinical factors relate to the risk of overuse injuries, and to investigate whether the relationships between potential risk factors and injury become stronger if injuries are grouped by location.

**Methods:**

The study is a prospective cohort study conducted during a Swedish track and field season over eleven months, from October to August. The cohort consisted of elite male and female track and field athletes competing in either middle- and long-distance running, sprinting, jumping, or throwing events (*n* = 96). Athletes performed a baseline screening at enrollment consisting of a clinical examination, running, and strength tests. Injury data was collected during the season by medical professionals and divided according to their anatomical location into upper-body, thigh/hip, knee, or foot/shank injuries.

**Results:**

Thirty-four (54.8%) injuries where located at the foot/shank, followed by sixteen injuries at the thigh/hip (25.8%). Only eight knee (12.9%) and four upper-body (6.5%) injuries were registered during the season and therefore not analysed. Effect sizes were calculated for all test variables. Small effect sizes (r_pb_ = .10–.23) were found for eleven risk factors between the overall injured (all injuries combined) and non-injured athletes. By further sub-grouping the injured group into thigh/hip injuries, effect size increased in hip adduction range of motion knee flexion velocity and the muscle flexibility of the iliopsoas. For foot/shank injuries, only the hamstring:quadriceps strength ratios increased for the right side to a small effect size.

**Conclusions:**

Injury grouping appears to increase effect size for certain risk factors. Athletes with a slower knee flexion velocity during stance phase were more likely to become injured (*p*-value <.03, r_pb_ = .37). An increased cohort size to further sub-divide injuries into specific diagnoses is needed.

## Background

Track and field is the collective name for a combination of sporting events, including middle- and long-distance running, sprint, jumping, and throwing events. The sport is governed by World Athletics representing over 200 different national member federations.

Previous research, regardless of subjects’ gender and age, has emphasized the high number of injuries in recreational and collegiate track and field [1–5], as well as elite track and field [6–8], with a consensus that the main cause of injury is overuse. At least 90% of all overuse injuries (OI) occur at the lower extremities, with a majority of OI occurring at the foot and thigh [1, 7–9]. There is, however, limited information on how OI develop in elite track and field. Athlete’s previous injury history, high training volume, and different biomechanical factors are assumed to be contributing risk factors [6, 7, 10, 11].

The main deficits of previous studies are their different methodological limitations. One common limitation is the cause-and-effect problem present in studies using a retrospective study design. These studies investigate differences between healthy and injured subjects, which can neither specify causes of, nor a compensatory effect of an injury. Instead, prospective study designs are considered essential to clarify cause-and-effect relationships and to determine interrelationships between different risk factors leading to OI [12–14].

Further limitations include small study populations that lead to statistical underpowering, and studies examining associations with OI solely from a biomechanical, clinical or training perspective. A consensus among researchers is that it is not a single discipline or variable causing OI, instead, the cause is multifactorial [15–17].

A typical way to evaluate injury risk in prospective studies is to compare the injured subjects to the non-injured subjects, independent from the location of injury [18]. It is questionable whether this general comparison is suitable to detect risk factors, as the mechanisms that cause OI might be different for different locations and diagnoses [19].

Understanding the different biomechanical and clinical mechanisms is crucial for the development and successful implementation of prevention strategies for elite track and field athletes. There are only a limited number of studies exploring biomechanical and clinical risk factors that have attempted to find associations with OI [1, 20–22], none of which involve elite track and field athletes.

The aim of this paper was therefore twofold: (1) to evaluate how biomechanical factors (movement patterns and strength) and clinical factors (muscle flexibility and joint range of motion) relate to the risk of OI in a cohort of elite Swedish track and field athletes, and (2) to investigate whether the relationships between the potential risk factors and injury become stronger if injuries are grouped by location.

## Methods

### Study design

The present study is based on a previously published study protocol [14], and is a prospective cohort study with participating athletes enrolled for a complete Swedish track and field season of eleven months (the 1:st of October through the end of August the following year). September was excluded for all athletes from all event groups, as most athletes in Sweden rest during this period.

All athletes underwent a baseline screening at enrollment consisting of a clinical examination, running analysis (except for throwers, high jumpers, and pole vaulters), and isometric strength tests. Injury data from all athletes were collected during the season.

The study was approved by the Regional Ethical Committee in Gothenburg (dnr 723–16) and follows the STROBE statement [23].

### Participants

The inclusion criteria was based on athlete performance; participating athletes had to have finished in the top six at the Swedish national championship or top three at the Swedish youth national championship or be enrolled at the elite track and field school in Gothenburg. All athletes had to be free from musculoskeletal pain or injury affecting their performance at their baseline screening as confirmed by the study’s physiotherapist following the clinical examination.

Recruitment of athletes was conducted together with Gothenburg’s Athletics Association (GFIF), which compiled a list of athletes eligible for inclusion. The study leader contacted all athletes on the list by e-mail or phone to invite them to partake in the study. Male and female athletes competing in middle- and long-distance running (800 m to 10,000 m), sprinting (60 m to 400 m including hurdle events), jumping (pole vault, high jump, long jump, and triple jump), and throwing events (shot put, javelin, hammer throw and discus) were recruited. A sample of 112 athletes enrolled at baseline. After the completion of the study period, 16 athletes were excluded from the data analysis and were considered dropouts, as they did not complete a full season due to personal reasons.

The final study population consisted of 96 athletes, divided into the event groups middle- and long-distance running (*n* = 33), sprinting (*n* = 30), jumping (*n* = 27), and throwing (*n* = 6) [Table 1].

**Table 1:** Characterization of the study population (mean values, SD in brackets) and break down of events (number of participants and % in brackets)

Study population	Female (***n*** = 44)	Male (***n*** = 52)	Total (n = 96)
Age (years)	20.7 (3.7)	19.2 (2.7)	19.9 (3.3)
Height (m)	1.71 (0.5)	1.84 (0.5)	1.78 (0.1)
Weight (kg)	61.1 (7.6)	74.3 (8.4)	68.2 (10.4)
BMI (kg/m^2^)	20.9 (2.1)	22.0 (2.2)	21.5 (2.2)
**Event group**			
M/L (n, %)	16, (48.5%)	17, (51.5%)	33, (100%)
Sprinting (n,)	11 (36.7%)	19 (63.3%)	30, (100%)
Jumping (n)	15 (55.6%)	12 (44.4%)	27, (100%)
Throwing (n)	2 (33.3%)	4 (66.6%)	6, (100%)

M/L = middle- and long-distance runners, n = number of athletes

### Clinical examination

All clinical examinations of the participating athletes were performed by the study’s physiotherapist according to the neutral-zero-method [24]. An inertial sensor, which included an accelerometer, a gyroscope, and a magnetic field sensor (SportMed A.G. SA, Bitburg, Germany), was used to measure passive joint range of motion and muscle flexibility. The software used were Mobee Fit and Mobee Med [25]. The inertial sensor was fixed with Velcro straps on the segment that was measured. Measurements of muscle flexibility were performed with athletes lying in a supine position for the hamstrings and iliopsoas, and in a prone position for the rectus femoris. Joint range of motion was measured for ankle dorsiflexion with athletes lying in a supine position. The maximum angular value from three repetitions on each side (left, right) was recorded. All athletes performed the hamstring test (*n* = 96), however, one athlete felt slight discomfort during the iliopsoas test and canceled the test (*n* = 95), and the throwers did not perform the rectus femoris and ankle dorsiflexion test (*n* = 90) as agreed upon with the elite coaches.

### Running analysis

The running analysis was carried out at a speed of 18 km/h on a treadmill (Rodby, RL 2500E × 700) in a laboratory setting with all athletes wearing the same type of standardized neutral running shoes. Thirty-two spherical reflective markers were placed on predetermined anatomical landmarks on all athletes according to International Sport Biomechanics (ISB) guidelines [26, 27]. Four segments were identified using the marker set: pelvis, thigh, shank and foot. For the pelvis two markers were attached on the anterior superior iliac spine and two markers on the posterior superior iliac spine. Three markers were attached on each thigh, positioned on the greater trochanter, and the lateral and medial femoral epicondyle. Six markers were attached on each shank, at the medial and lateral ridge of tibial plateau, tibial tuberosity, medial crest of tibia, medial and lateral malleolus and five markers to each foot on the subjects’ footwear positioned on the lateral, medial and posterior heel counter, the fifth metatarsal, and the tip of the shoe. The 3D software Qualisys Track Manager with 16 infra-red cameras (Qualisys, Gothenburg, Sweden) was used to capture data. All data was captured at a rate of 400 Hz. The analysis of joint motion was restricted to the full stance phase which was detected by using an algorithm described by Maiwald et al. (2009) and further implemented by Hein et al. (2014) and Jungmalm et al. (2020) [13, 18, 28].

The following movement variables were evaluated during stance: hip adduction range of motion, initial knee flexion angle, knee flexion range of motion, knee flexion velocity, ankle eversion range of motion, ankle eversion velocity, and initial ankle flexion angle. The chosen movement variables have previously been evaluated in other study populations (e.g. recreational runners) with varying levels of evidence to injury, but no information exists for elite athletics athletes [13, 29, 30].

Motions of the hip, knee, and ankle joints were calculated relative to the static neutral standing position. The results were based on 10 consecutive strides for each side (left, right), and were analysed through custom-written MATLAB (MathWorks Inc., Natick, MA) code, and reported as averages [13, 18].

Only middle- and long-distance runners, sprinters, long jumpers, and triple jumpers performed the running analysis (*n* = 81).

### Isometric strength tests

The baseline strength tests consisted of isometric maximal voluntary contractions in six different measurement devices (David Health Solutions Ltd., Helsinki, Finland). The strength tests consisted of hip adduction and hip abduction (bilateral) (15°), knee flexion and knee extension (unilateral) (30°), abdominal flexion (0°) and extension of the trunk (30°), as well as trunk rotation (unilateral) (±30°). All athletes had two trials, a third if the difference was more than 10% with the highest value noted. All measurements were performed according to a standardized test protocol and normalized to bodyweight [14]. All athletes were seated and secured with a safety belt, and no self-stabilization was permitted during the trials. Calculations for the following strength balance ratios were performed; trunk flexion:extension, trunk rotation right:left, hip abduction:adduction, and knee flexion:extension. All athletes performed the isometric strength tests, however, one athlete experienced discomfort during the hip adduction test (*n* = 95). Furthermore, two athletes experienced discomfort during the knee flexion test and therefore discontinued their tests (*n* = 94).

### Injury data collection

All injury data was collected by two medical professionals as described in previous studies [6, 14]. An injury was defined *“as any musculoskeletal pain felt during athletics training or competition that inflicted a non-voluntary reduction of or complete stop from athletics training for at least 24 h, and was diagnosed by a trained medical professional, e.g. a physiotherapist and/or sports physician”* [6, 14]. Injuries were categorized according to their onset; gradual or sudden onset caused by overuse [31]. No recurrent or traumatic (acute) injuries were recorded, as the recommendation from previous research has been to focus solely on OI [32]. Injury data for athletes enrolled at the elite track and field school was collected by the physiotherapist employed by the school according to the same injury definition and classification and subsequently added to the dataset. For athletes that sustained multiple injuries during the season, only the first injury sustained after the baseline screening was included in the analyses. Injury diagnoses were grouped according to their anatomical locations into thigh/hip, upper-body, knee, and foot/shank injuries.

### Statistical analysis

All analyses were conducted using JASP statistics (JASP Version 0.14). The analyses were conducted in two steps. In a first step, conducting Mann-Whitney U-tests, the differences in the study variables between injured and non-injured athletes (without any specification of injury location) were investigated. The reasons for conducting Mann-Whitney U-tests, in comparison to independent t-tests were: (a) the small sample size, and (b) non-normality within many of the study variables. Study variables were selected in pairs for further analysis if either left or right side showed at least a small effect size (r_pb_ = .10). We calculated Rank-Biserial correlations (with accompanying 95% Confidence Intervals) in line with previous recommendations [33]. To interpret the effect sizes, we followed the recommendation from McGrath et al. (2006) suggesting three categories: small (r_pb_ = .10), medium (r_pb_ = .24) and large (r_pb_ = .37) [34]. In a second step, differences between injured (specific injury locations) and non-injured athletes (with regard to specific injury locations) were researched. For the Mann-Whitney U-tests, a *p*-value <.05 was considered to indicate statistically significant results. Due to the low number of overuse injuries in the upper-body and at the knee, differences were only investigated for thigh/hip and foot/shank injuries.

### Results

Overall, sixty-two out of ninety-six athletes (64.6%) were injured. Not all injured athletes performed the movement screening test as displayed in Table 2. The distribution of injuries with regard to location for the different screening procedures are also listed in Table 2. For all screening procedures, the foot/shank injury location showed the highest frequency of injured athletes followed by the thigh/hip injury location.

**Table 2:** Number of injured athletes with regard to injury location for the three different screening protocols

	Movement (n = 81)	Clinical (*n* = 95)	Strength (n = 95)
Overall injured	55, (100%)	62, (100%)	62, (100%)
Thigh/hip injured	15, (27%)	17, (28%)	16, (26%)
Upper-body injured	4, (7%)	4, (6%)	4, (6%)
Knee injured	5, (9%)	7, (11%)	8, (13%)
Foot/shank injured	31, (57%)	34, (55%)	34, (55%)

The first analyses looking at the potential differences between injured (all injuries combined) and non-injured athletes showed only one statistically significant difference (ankle eversion velocity left). There were small effect sizes (r_pb_ = .10–.23) for eleven biomechanical and clinical variables [Table 3].

**Table 3:** Comparison of injured and non-injured athletes for movement, clinical and strength variables

	INJ.	NON-INJ.	INJ. vs NON-INJ.	
	*m ± SD*	*m ± SD*	*p*	*r*_*pb*_
Movement				
HAD_ROM_R [°]_	5.38 [2.58]	6.03 [2.53]	.26	.16
HAD_ROM_L [°]_	4.80 [2.35]	5.50 [2.44]	.14	.20
KF_TIM_R [%]_	15.45 [6.98]	15.37 [5.61]	1.0	.00
KF_TIM_L [%]_	15.51 [8.02]	16.10 [8.01]	.74	.06
KF_ROM_R [°]_	26.34 [6.17]	25.63 [6.45]	.63	−.07
KF_ROM_L [°]_	26.72 [6.20]	26.02 [7.12]	.86	−.02
KF_VEL_R [°/s]_	327.27 [132.87]	371.77 [185.00]	.67	.06
KF_VEL_L [°/s]_	334.78 [129.59]	379.44 [169.10]	.44	.11
AEV_TIM_R [%]_	10.50 [7.57]	9.71 [6.82]	.81	−.03
AEV_TIM_L [%]_	11.11 [7.63]	10.29 [6.48]	.94	−.01
AEV__R [°]_	15.04 [6.26]	16.81 [9.16]	.58	.08
AEV__L [°]_	14.72 [6.30]	17.43 [8.37]	.17	.19
AEV_VEL_R [°/s]_	250.09 [120.81]	287.86 [135.23]	.18	.19
AEV_VEL_L [°/s]_	242.45 [119.76]	298.40 [135.10]	.05*	.27
Clinical				
ADF_ROM_R [°]_	26.21 [8.20]	27.31 [6.21]	.56	.08
ADF_ROM_L [°]_	27.07 [8.30]	25.79 [4.55]	.91	−.02
HAM_FLEX_R [°]_	92.97 [13.09]	92.15 [12.33]	.99	−.00
HAM_FLEX_L [°]_	86.82 [14.15]	86.79 [11.96]	.79	.03
RF_FLEX_R [°]_	139.02 [16.63]	144.38 [7.48]	.26	.15
RF_FLEX_L [°]_	148.62 [8.45]	150.38 [11.27]	.65	.06
IL_FLEX_R [°]_	108.68 [11.73]	105.70 [8.45]	.24	−.15
IL_FLEX_L [°]_	108.92 [8.38]	106.27 [9.13]	.14	−.18
Strength				
HAB:HAD _[Nm/kg]_	0.79 [0.19]	0.84 [0.19]	.15	.18
TF:TE _[Nm/kg]_	0.59 [0.18]	0.56 [0.15]	.76	−.04
TR_[Nm/kg]_	1.02 [0.14]	1.04 [0.17]	.63	.06
H:Q__R [Nm/kg]_	0.83 [0.16]	0.84 [0.16]	.93	.01
H:Q__L [Nm/kg]_	0.82 [0.13]	0.83 [0.17]	.42	−.10

*HAD*_*ROM*_ Hip adduction range of motion, *KF*_*TIM*_ Timing of maximal knee flexion, *KF*_*ROM*_ Knee flexion range of motion, *KF*_*VEL*_ Knee flexion velocity, *AEV*_*TIM*_ Timing of maximal ankle eversion, *AEV* Ankle eversion, *AEV*_*VEL*_ Ankle eversion velocity, *ADF*_*ROM*_ Ankle dorsiflexion range of motion, *HAM*_*FLEX*_ Hamstring flexibility, *RF*_*FLEX*_ Rectus femoris flexibility, *IL*_*FLEX*_ Iliopsoas flexibility, *HAB:HAD* Hip abduction:adduction strength ratio, *TF:TE* Trunk flexion:extension ratio, *TR* Trunk rotation strength, *H:Q* Hamstring:quadriceps strength ratio, *R* Right side, *L* Left side, *m* Mean, *SD* Standard deviation, * indicates *p* ≤ 0.05, *r*_*pb*_ Rank-Biserial Correlation

For the second step analyses, comparing thigh/hip injured athletes with non- thigh/hip injured athletes showed only one statistically significant difference (knee flexion velocity left). There were, however, a substantially increase in effect size for knee flexion velocity for both left (from r_pb_ = .11 to r_pb_ = .37) and right side (from r_pb_ = .06 to r_pb_ = .29). Hip adduction range of motion (both sides) and iliopsoas flexibility (both sides) also increased in effect size but were rated as not practically relevant. There were no statistically significant results for the comparison of the foot/shank injured athlete to the non-foot/shank injured athletes. A decrease in effect size was seen for all variables when comparing the foot/shank injured athletes to the non- foot/shank injured athletes [Table 4]. The only exception was for the H:Q strength ratio on the right side which increased to small effect size (from r_pb_ = .01 to r_pb_ = −.11).

**Table 4:** Comparison of injured and non-injured athletes (overall, thigh/hip and foot/shank) for movement, clinical and strength variables

	OA INJ.	OA NON-INJ.			T/H INJ.	T/H NON-INJ.			F INJ.	F NON-INJ.		
	*m ± SD*	*m ± SD*	*p*	*r*_*pb*_	*m ± SD*	*m ± SD*	*p*	*r*_*pb*_	*m ± SD*	*m ± SD*	*p*	*r*_*pb*_
Movement												
HAD_ROM_R [°]_	5.38 [2.58]	6.03 [2.53]	.26	.16	4.68 [2.38]	5.80 [2.58]	.16	.24	5.87 [2.71]	5.42 [2.48]	.60	−.07
HAD_ROM_L [°]_	4.80 [2.35]	5.50 [2.44]	.14	.20	4.19 [2.01]	5.21 [2.44]	.13	.25	5.33 [2.54]	4.83 [2.29]	.54	−.08
KF_VEL_R [°/s]_	327.27 [132.87]	371.77 [185.00]	.67	.06	257.82 [41.53]	360.59 [161.28]	.08	.29	336.10 [129.64]	344.94 [165.26]	.84	−.03
KF_VEL_L [°/s]_	334.78 [129.59]	379.44 [169.10]	.44	.11	266.14 [42.50]	367.97 [152.16]	.03*	.37	341.39 [125.37]	353.91 [155.34]	.91	−.02
AEV__R [°]_	15.04 [6.26]	16.81 [9.16]	.58	.08	14.86 [5.61]	15.78 [7.66]	.70	−.07	15.33 [6.66]	15.78 [7.74]	.74	.05
AEV__L [°]_	14.72 [6.30]	17.43 [8.37]	.17	.19	15.24 [5.06]	15.67 [7.51]	.53	−.11	14.78 [6.42]	16.09 [7.50]	.38	.12
AEV_VEL_R [°/s]_	250.09 [120.81]	287.86 [135.23]	.18	.19	245.12 [88.32]	266.10 [133.35]	.88	−.03	257.43 [138.04]	265.17 [119.32]	.45	.10
AEV_VEL_L [°/s]_	242.45 [119.76]	298.40 [135.10]	.05*	.27	250.26 [84.70]	262.72 [134.95]	.64	−.08	245.74 [124.95]	269.51 [128.32]	.30	.14
Clinical												
RF_FLEX_R [°]_	139.02 [16.63]	144.38 [7.48]	.26	.15	140.77 [8.48]	140.74 [15.62]	.30	.16	139.15 [19.68]	141.67 [10.51]	.63	−.06
RF_FLEX_L [°]_	148.62 [8.45]	150.38 [11.27]	.65	.06	148.94 [5.80]	149.25 [10.11]	.82	.04	148.64 [10.20]	149.51 [9.03]	.81	−.03
IL_FLEX_R [°]_	108.68 [11.73]	105.70 [8.45]	.24	−.15	108.77 [11.46]	107.40 [10.66]	.17	−.21	107.85 [11.74]	107.53 [10.26]	.78	.04
IL_FLEX_L [°]_	108.92 [8.38]	106.27 [9.13]	.14	−.18	111.25 [8.61]	107.42 [8.65]	.15	−.23	108.38 [8.58]	107.79 [8.81]	.68	−.05
Strength												
HAB:HAD _[Nm/kg]_	0.79 [0.19]	0.84 [0.19]	.15	.18	0.76 [0.08]	0.82 [0.21]	.25	.15	0.77 [0.16]	0.83 [0.21]	.39	.11
H:Q__R [Nm/kg]_	0.83 [0.16]	0.84 [0.16]	.93	.01	0.79 [0.15]	0.84 [0.16]	.62	.08	0.84 [0.15]	0.83 [0.17]	.39	−.11
H:Q__L [Nm/kg]_	0.82 [0.13]	0.83 [0.17]	.42	−.10	0.80 [0.12]	0.83 [0.15]	.84	.03	0.83 [0.13]	0.82 [0.15]	.60	−.07

*HAD*_*ROM*_ Hip adduction range of motion, *KF*_*VEL*_ Knee flexion velocity, *AEV* Ankle eversion, *AEV*_*VEL*_ Ankle eversion velocity, *RF*_*FLEX*_ Rectus femoris flexibility, *IL*_*FLEX*_ Iliopsoas flexibility, *HAB:HAD* Hip abduction:adduction strength ratio, *H:Q* Hamstring:quadriceps strength ratio, *OA INJ Overall injured, OA NON-INJ Overall non-injured, T/H INJ* Thigh/hip injured, *T/H NON-INJ* Thigh/hip non-injured, *R* Right side, *L* Left side, *m* Mean, *SD* Standard deviation, * indicates *p* ≤ 0.05, *r*_*pb*_ Rank-Biserial Correlation

The revealed differences between athletes with and without thigh/hip injuries show that the injured athletes had a lower hip adduction range of motion (left side M = 4.19° compared to M = 5.21° and right side M = 4.68° compared to M = 5.80°), slower knee flexion velocity (left side M = 266.14°/s compared to M = 367.97°/s and right side M = 257.82°/s compared to M = 360.59°/s), and weaker hip abductors (M = 0.76 Nm/kg compared to 0.82 Nm/kg) [Table 5].

**Table 5:** Movement, clinical and strength variables in athletes with thigh/hip injuries and athletes without thigh/hip injuries

	*Group*	*n*	*p*	*r*_*pb*_	*m*	*SD*
Movement						
HAD_ROM_R [°]_	Inj	15	.16	.24	4.68	2.38
	No	66			5.80	2.58
HAD_ROM_L [°]_	Inj	15	.13	.25	4.19	2.01
	No	66			5.21	2.44
KF_VEL_R [°/s]_	Inj	15	.08	.29	257.82	41.53
	No	66			360.59	161.28
KF_VEL_L [°/s]_	Inj	15	.03*	.37	266.14	42.50
	No	66			367.97	152.16
AEV__R [°]_	Inj	15	.70	−.07	14.86	5.61
	No	66			15.78	7.66
AEV__L [°]_	Inj	15	.53	−.11	15.24	5.06
	No	66			15.67	7.51
AEV_VEL_R [°/s]_	Inj	15	.88	−.03	245.12	88.32
	No	66			266.10	133.35
AEV_VEL_L [°/s]_	Inj	15	.64	−.08	250.26	84.70
	No	66			262.72	134.95
Clinical						
RF_FLEX_R [°]_	Inj	17	.30	.16	140.77	8.48
	No	73			140.74	15.62
RF_FLEX_L [°]_	Inj	17	.82	.04	148.94	5.80
	No	73			149.25	10.11
IL_FLEX_R [°]_	Inj	17	.17	−.21	108.77	11.46
	No	78			107.40	10.66
IL_FLEX_L [°]_	Inj	17	.15	−.23	111.25	8.61
	No	78			110.65	8.65
Strength						
HAB:HAD _[Nm/kg]_	Inj	16	.25	.15	0.76	0.08
	No	79			0.82	0.21
H:Q__R [Nm/kg]_	Inj	17	.62	.08	0.79	0.15
	No	77			0.84	0.16
H:Q__L [Nm/kg]_	Inj	17	.84	.03	0.80	0.12
	No	77			0.83	0.15

*HAD*_*ROM*_ Hip adduction range of motion, *KF*_*VEL*_ Knee flexion velocity, *AEV* Ankle eversion, *AEV*_*VEL*_ Ankle eversion velocity, *RF*_*FLEX*_ Rectus femoris flexibility, *IL*_*FLEX*_ Iliopsoas flexibility, *HAB:HAD* Hip abduction:adduction strength ratio, *H:Q* Hamstring:quadriceps strength ratio, *R* Right side, *L* Left side, *n* number, * indicates p ≤ 0.05, *r*_*pb*_ Rank-Biserial Correlation, *m* Mean, *SD* Standard deviation, *95% CI* Confidence interval

## Discussion

The overall number of athletes with injuries to the foot and shank in our study correspond with the results from Jacobsson et al. [7]. However, our cohort had substantially more athletes with injuries to the thigh and hip.

For the overall evaluation between injured and non-injured athletes, small effect sizes were identified for a number of variables from the baseline screening, which lead us to suspect a possible relationship between certain biomechanical and clinical risk factors, and OI.

Comparing the non-injured thigh/hip group to athletes with thigh/hip injuries, an increase in effect size was seen for variables that more directly analyze thigh/hip movement, and thigh/hip flexibility. Remote variables (e.g. ankle movement) did not increase. A possible explanation could be that the measures that increased in effect size are closer to the injury location, and thus reflect injury patterns better than remote variables.

The injury group of athletes with foot/shank injuries saw a decrease in effect sizes for all measurement variables, except for the H:Q strength ratio for the right side compared to the non-injured foot/shank group. A reason for this could be that there were more than double the amount of injury diagnoses in the foot/shank-injured group compared to thigh/hip-injured group, making the foot/shank-injured group more inhomogeneous with regard to possible underlying injury patterns. The evidence on the risk between movement variables such as ankle eversion and running related injuries has been described as inconsistent in the past, and assumed to be largely dependent on the study population and type of injury being studied [35, 36]. This is in accordance with our findings.

While running, knee flexion velocity was slower in the thigh/hip-injured group compared with the thigh/hip non-injured group. This would suggest that impact cushioning via knee flexion is delayed. As we saw that knee flexion ROM was the same for thigh/hip injured and thigh/hip non-injured, a possible explanation is that the thigh/hip-injured group had weaker hip flexors (iliopsoas) or a decreased flexibility of the gluteus maximus. Neither were measured in the current study.

The possible lower HAB:HAD strength ratio in the thigh/hip injured group would indicate that the hip abductors were weaker compared to the hip adductors. This is in accordance with previous studies, where weak hip abductors have been discussed as a risk factor in the development of running-related injuries, especially for hip injuries [18, 37].

The comprehensive baseline screening, the prospective study design, and the use of medical professionals to diagnose injuries instead of the commonly used self-report approach, which has previously been shown to be inconsistent [38, 39], are the major strengths of the present study. A limitation of the study is the relatively low number of participants. The small cohort size makes it impossible to sub-divide athletes according to gender and specific event group, and the injury locations used are still a rough injury location grouping. To be more exact, the evaluation should be done for each diagnosis separately. Another possible limitation could be that we have been comparing athletes with thigh/hip injuries to thigh/hip non-injured athletes (and not completely uninjured athletes). This might have affected a more precise distinction between thigh/hip injured and non-injured athletes. The study’s findings can only be generalized to similar elite track and field athletes.

## Conclusions

In running, athletes with a slower knee flexion velocity during stance phase were more likely to become injured. This indicates impact cushioning via knee flexion is delayed. As knee flexion ROM was the same for thigh/hip injured and thigh/hip non-injured, the thigh/hip-injured group might have weaker hip flexors or a decreased flexibility of the gluteus maximus. There is a need for large cohort sizes in prospective studies to further sub-divide athletes according to gender, event group, and specific injury diagnosis.

## Data Availability

The datasets used and/or analyzed during the current study are available from the corresponding author on reasonable request.

## Abbreviations

*BMI*: Body mass index
*CI*: Confidence Interval
*GFIF*: Gothenburg Athletics Association
*HAB*: Hip abduction
*HAD*: Hip adduction
*H:Q*: Hamstring:Quadriceps
*ISB*: International Sports Biomechanics
*M/L*: Middle- and long-distance runner
*OI*: Overuse injuries
*ROM*: Range of motion
*SD*: Standard deviation

## References

[1] Bennell KL, Crossley K (1996). Musculoskeletal injuries in track and field: incidence, distribution and risk factors. Aust J Sci Med Sport.

[2] D'souza D (1994). Track and field athletics injuries--a one-year survey. Br J Sports Med.

[3] Edouard P, Depiesse F, Serra J-M. Throwing arm injuries in high-level athletics throwers. Sci Sports. 2010;25(6):318–22. 10.1016/j.scispo.2010.08.004.

[4] Lysholm J, Wiklander J (1987). Injuries in runners. Am J Sports Med.

[5] Tyflidis A, Kipreos G, Tripolitsioti AD, Stergioulas AT. Epidemiology of track & field injuries: a one year experience in athletic schools. Biol Sport. 2012;29(4):291.

[6] Zachrisson AL, Ivarsson A, Desai P, Karlsson J, Grau S (2020). Athlete availability and incidence of overuse injuries over an athletics season in a cohort of elite Swedish athletics athletes-a prospective study. Inj Epidemiol.

[7] Jacobsson J, Timpka T, Kowalski J, Nilsson S, Ekberg J, Dahlström Ö, Renström PA (2013). Injury patterns in Swedish elite athletics: annual incidence, injury types and risk factors. Br J Sports Med.

[8] Ahuja A, Ghosh A (1985). Pre-Asiad'82 injuries in elite Indian athletes. Br J Sports Med.

[9] Edouard P, Alonso J-M. Epidemiology of track and field injuries. New Studies in Athletics. 2013;28(1/2):85–92.

[10] Malliaropoulos N, Mendiguchia J, Pehlivanidis H, Papadopoulou S, Valle X, Malliaras P, Maffulli N (2012). Hamstring exercises for track and field athletes: injury and exercise biomechanics, and possible implications for exercise selection and primary prevention. Br J Sports Med.

[11] Malliaropoulos N, Bikos G, Meke M, Vasileios K, Valle X, Lohrer H, Maffulli N, Padhiar N (2018). Higher frequency of hamstring injuries in elite track and field athletes who had a previous injury to the ankle-a 17 years observational cohort study. J Foot Ankle Res.

[12] Nielsen RO, Buist I, Parner ET, Nohr EA, Sørensen H, Lind M, Rasmussen S (2013). Predictors of running-related injuries among 930 novice runners: a 1-year prospective follow-up study. Orthop J Sports Med.

[13] Hein T, Janssen P, Wagner-Fritz U, Haupt G, Grau S (2014). Prospective analysis of intrinsic and extrinsic risk factors on the development of a chilles tendon pain in runners. Scand J Med Sci Sports.

[14] Zachrisson AL, Desai P, Karlsson J, Johanesson E, Grau S (2018). Overuse injuries in Swedish elite athletics–a study protocol for a prospective multifactorial cohort study. BMC Musculoskelet Disord.

[15] Winter SC, Gordon S, Brice SM, Lindsay D, Barrs S (2020). A multifactorial approach to overuse running injuries: a 1-year prospective study. Sports Health.

[16] Wen DY (2007). Risk factors for overuse injuries in runners. Curr Sports Med Rep.

[17] Hreljac A (2005). Etiology, prevention, and early intervention of overuse injuries in runners: a biomechanical perspective. Phys Med Rehabil Clin N Am.

[18] Jungmalm J, Nielsen RØ, Desai P, Karlsson J, Hein T, Grau S (2020). Associations between biomechanical and clinical/anthropometrical factors and running-related injuries among recreational runners: a 52-week prospective cohort study. Inj Epidemiol.

[19] Grau S, Maiwald C, Krauss I, Axmann D, Horstmann T (2008). The influence of matching populations on kinematic and kinetic variables in runners with iliotibial band syndrome. Res Q Exerc Sport.

[20] Knapik JJ, Bauman CL, Jones BH, Harris JM, Vaughan L (1991). Preseason strength and flexibility imbalances associated with athletic injuries in female collegiate athletes. Am J Sports Med.

[21] Hreljac A, Marshall RN, Hume PA (2000). Evaluation of lower extremity overuse injury potential in runners. Med Sci Sports Exerc.

[22] Malliaropoulos N, Papacostas E, Kiritsi O, Rad P-M, Papalada A, Gougoulias N, Maffulli N (2010). Posterior thigh muscle injuries in elite track and field athletes. Am J Sports Med.

[23] Vandenbrouckel JP, von Elm E, Altman DG, Gotzsche PC, Mulrow CD, Pocock SJ (2007). Strengthening the reporting of observational studies in epidemiology (STROBE): explanation and elaboration. PLoS Med.

[24] Ryf C, Weymann A (1995). The neutral zero method—a principle of measuring joint function. Injury..

[25] Kraus K, Kraus E, Gojanovic B, Fourchet F. Concurrent Validity of 2D and Inertial Goniometer Motion Assessment. Int J Athletic Therapy Training. 2019;1(aop):1–6.

[26] Wu G, Siegler S, Allard P, Kirtley C, Leardini A, Rosenbaum D, Whittle M, D'Lima DD, Cristofolini L, Witte H, Schmid O, Stokes I, Standardization and Terminology Committee of the International Society of Biomechanics (2002). ISB recommendation on definitions of joint coordinate system of various joints for the reporting of human joint motion—part I: ankle, hip, and spine. J Biomech.

[27] Wu G, Van der Helm FC, Veeger HD, Makhsous M, Van Roy P, Anglin C (2005). ISB recommendation on definitions of joint coordinate systems of various joints for the reporting of human joint motion—part II: shoulder, elbow, wrist and hand. J Biomech.

[28] Maiwald C, Sterzing T, Mayer T, Milani T. Detecting foot-to-ground contact from kinematic data in running. Footwear Sci. 2009;1(2):111–8. 10.1080/19424280903133938.

[29] Kuhman DJ, Paquette MR, Peel SA, Melcher DA (2016). Comparison of ankle kinematics and ground reaction forces between prospectively injured and uninjured collegiate cross country runners. Hum Mov Sci.

[30] Noehren B, Hamill J, Davis I (2013). Prospective evidence for a hip etiology in patellofemoral pain. Med Sci Sports Exerc.

[31] Timpka T, Alonso J-M, Jacobsson J, Junge A, Branco P, Clarsen B, Kowalski J, Mountjoy M, Nilsson S, Pluim B, Renström P, Rønsen O, Steffen K, Edouard P (2014). Injury and illness definitions and data collection procedures for use in epidemiological studies in athletics (track and field): consensus statement. Br J Sports Med.

[32] Alonso J-M, Edouard P, Fischetto G, Adams B, Depiesse F, Mountjoy M (2012). Determination of future prevention strategies in elite track and field: analysis of Daegu 2011 IAAF championships injuries and illnesses surveillance. Br J Sports Med.

[33] Ivarsson A, Andersen MB, Johnson U, Lindwall M (2013). To adjust or not adjust: nonparametric effect sizes, confidence intervals, and real-world meaning. Psychol Sport Exerc.

[34] McGrath RE, Meyer GJ (2006). When effect sizes disagree: the case of r and d. Psychol Methods.

[35] Munteanu SE, Barton CJ (2011). Lower limb biomechanics during running in individuals with achilles tendinopathy: a systematic review. J Foot Ankle Res.

[36] Ceyssens L, Vanelderen R, Barton C, Malliaras P, Dingenen B. Biomechanical risk factors associated with running-related injuries: a systematic review. Sports Med. 2019;49(7):1095–115. 10.1007/s40279-019-01110-z 31028658

[37] Powers CM (2010). The influence of abnormal hip mechanics on knee injury: a biomechanical perspective. J Orthop Sports Phys Ther.

[38] Smits D-W, Backx F, Van Der Worp H, Van Middelkoop M, Hartgens F, Verhagen E (2019). Validity of injury self-reports by novice runners: comparison with reports by sports medicine physicians. Res Sports Med.

[39] Gabbe BJ, Finch CF, Bennell KL, Wajswelner H (2003). How valid is a self reported 12 month sports injury history?. Br J Sports Med.

